# Cost-effectiveness of pembrolizumab versus chemotherapy in patients with platinum-pretreated, recurrent or metastatic nasopharyngeal cancer

**DOI:** 10.1186/s12962-024-00515-6

**Published:** 2024-01-24

**Authors:** Jing Nie, Huina Wu, Qian Wu, Lihui Liu, Ke Tang, Shuo Wang, Jiyong Wu

**Affiliations:** 1 Department of Pharmacy, Shandong Second Provincial General Hospital, Jinan, Shandong China; 2 College of Pharmacy, Shandong Medical College, Jinan, Shandong China

**Keywords:** Nasopharyngeal cancer, PD-1, Pembrolizumab, Chemotherapy, Capecitabine, Gemcitabine, Docetaxel, Cost-effectiveness

## Abstract

**Background:**

Programmed cell death protein 1 (PD-1) monoclonal antibody, pembrolizumab, is a promising drug for platinum-pretreated, recurrent or metastatic nasopharyngeal cancer (NPC). We aimed to assess the cost-effectiveness of pembrolizumab compared with chemotherapy for Chinese patients in this NPC.

**Methods:**

The cost-effectiveness of pembrolizumab versus chemotherapy was evaluated using a partitioned survival model with a 5-year boundary. Efficacy and toxicity data were derived from the KEYNOTE-122 trials. Economic indicators including life-years (LYs), quality-adjusted life-years (QALYs), incremental cost-effectiveness ratio (ICER), and lifetime cost were used. One-way analysis and probabilistic sensitivity analysis (PSA) were performed to explore the uncertainties. Additionally, various scenario analyses, including different pembrolizumab price calculations and discount rates were performed.

**Results:**

Pembrolizumab or chemotherapy alone respectively yielded 2.82 QALYs (3.96 LYs) and 2.73 QALYs (3.93 LYs) with an ICER of $422,535 per QALYs ($1,232,547 per LYs). This model was primarily influenced by the price of pembrolizumab. Furthermore, PSA indicated that pembrolizumab had none probability of being cost-effective compared with chemotherapy at a willingness-to- pay (WTP) of $38223. Scenario analyses revealed that irrespective of any potential price reduction or adjustments in the discount rate, no discernible impact on the ultimate outcome was observed.

**Conclusion:**

Pembrolizumab was less cost-effective for patients with platinum-pretreated, recurrent or metastatic NPC compared with chemotherapy in China.

**Supplementary Information:**

The online version contains supplementary material available at 10.1186/s12962-024-00515-6.

## Introduction

Nasopharyngeal carcinoma (NPC) is a malignancy arising from the malignant epithelial cells in the nasopharyngeal cavity, specifically on the top and side walls. This type of cancer is characterized by its highly invasive nature and exhibits substantial variation in incidence rates across different regions worldwide. Notably, Southeast Asian countries, particularly China, bear a high burden of NPC cases. Men are more susceptible to developing NPC compared to women, and the prognosis for women is generally more favorable [1, 2]. In 2020, the International Agency for Research on Cancer (IARC) reported a global incidence of 133,354 new cases of NPC, leading to 80,008 deaths. China alone accounted for a staggering 46% of the total new cases worldwide, underscoring the significant threat NPC poses to human life and well-being [3]. Nevertheless, encouragingly, there has been a decline in the incidence rate and a substantial reduction in mortality over the past decade, indicating a positive epidemiological trend.

NPC is frequently diagnosed at advanced stage [4], necessitating a multi-modal approach to treatment involving radiotherapy and chemotherapy [5]. However, this treatment regimen is often protracted and accompanied by persistent adverse reactions. Additionally, the available standard first-line treatment options for patients with recurrent or metastatic NPC are limited. Typically, a combination of platinum-based chemotherapy drugs is administered. Despite this approach, the median progression-free survival (PFS) time is only about 7 months, with a less than 20% five-year overall survival (OS) rate [6].

Pembrolizumab, a monoclonal antibody that targeting the programmed cell death 1 (PD-1) receptor and blocking its interaction with programmed cell deathligand 1 (PD-L1) and PD-L2, shows great potential as an effective immunotherapeutic treatment [7]. By restoring tumor-specific T cell immunity through blocking the PD-1 pathway, this therapy holds promise in combating NPC. Pembrolizumab has received extensive global approval as an immune checkpoint inhibitor (ICI), with 17 indications across 11 tumor types. It has been approved for use in patients with recurrent or metastatic head and neck squamous cell carcinoma who have previously undergone platinum-based chemotherapy regimens. The Chinese Society of Clinical Oncology’s guidelines for NPC diagnosis and treatment recommend chemotherapy as the preferred option for patients with recurrent or metastatic NPC [8]. Specifically, cisplatin plus gemcitabine is commonly employed as the first-line systemic treatment for unresectable recurrent or metastatic NPC [9].

The effectiveness of pembrolizumab was demonstrated in patients with recurrent or metastatic NPC who had previously received extensive treatment, as evidenced by the KEYNOTE-028 study [10]. Subsequently, the KEYNOTE-122 study was conducted to further investigate the comparative efficacy of pembrolizumab versus chemotherapy in these patients. Randomly assigned participants receive either pembrolizumab or chemotherapy. The study observed a median OS of 17.2 months in the pembrolizumab group and 15.3 months in the chemotherapy group, while the median progression-free survival (PFS) was 4.1 months versus 5.5 months, respectively. Although pembrolizumab did not yield significant improvements in OS, it exhibited favorable tolerability with a lower incidence of treatment-related adverse events [9]. Against this backdrop, our study aims to assess the cost-effectiveness of pembrolizumab versus chemotherapy in platinum-pretreated, recurrent, or metastatic NPC, considering the perspective of Chinese healthcare system.

## Materials and methods

### Model construction

**Fig. 1:**
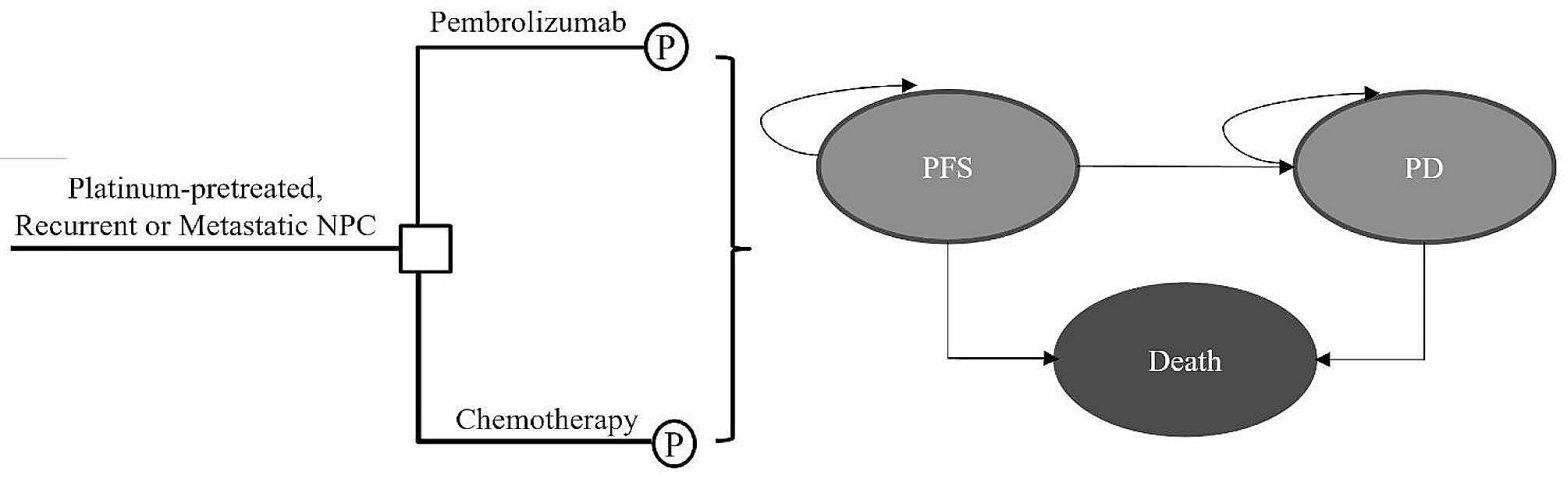
Model structure for Platinum-pretreated, Recurrent or Metastatic NPC. NPC, Nasopharyngeal carcinoma; P, Partitioned survival model; PFS, progression-free survival; PD, progressed disease

A partitioned survival model (PSM) was established to evaluate the economic and treatment efficacy for platinum-pretreated patients with recurrent or metastatic NPC (Fig. 1), comparing pembrolizumab with chemotherapy. The model considers three mutually exclusive health state: PFS (as the starting state for all patients), progressed disease (PD), and death. A simulated hypothetical cohort with similar characteristics to those in the KEYNOTE-122 clinical trial was used [9]. Eligible patients included those aged 18 years or older with histologically confirmed NPC who had received at least one prior platinum-based chemotherapy. The assumption was made that all patients began in the PFS state, and at the end of each cycle, they either remained in this state or transitioned to a health state of deteriorating severity. Patients who transitioned from PFS to PD were unable to revert back to their PFS status, instead exhibiting continued disease progression or succumbing to mortality. The therapeutic regimen replicated that of the KEYNOTE-122 trials. Patients were randomized to receive either pembrolizumab (200 mg administered per treatment cycle), or chemotherapy, which consisted of capecitabine (1000 mg/m^2^ orally twice daily on days 1–14 per cycle), gemcitabine (1250 mg/m^2^ intravenously twice per cycle), or docetaxel (75 mg/m^2^ intravenously once per cycle). The cost of chemotherapy was determined using a base-case body surface area (BSA) value of 1.72 m^2^ [11, 12].

We utilized the GetData Graph Digitizer (version 2.20) to extract data points from both PFS and OS curves, incorporating the outcomes of the KEYNOTE-122 trial. Subsequently, the data were subjected to modeling using several parametric survival functions, namely weibull, log-logistic, exponential, log-normal, gompertz, and generalized gamma. To determine the most suitable function for each survival curve, we employed the Akaike information criterion (AIC) and Bayesian information criterion (BIC), wherein lower values indicated a superior fit to the data. Our analysis determined that the lognormal distribution provided best fit for the PFS data, while the exponential distribution was suitable for extrapolating the OS for all groups (see Supplementary Appendix). Consequently, we used the lognormal and exponential distributions to calculate transition probabilities. The parametric survival curves were generated using the RStudio 2022.02.0 software, and PSM model was constructed using TreeAge Pro 2022. To estimate the transition probability from PFS to death, we incorporated the general population mortality rates obtained from the mortality tables of the resident population in 2022, as published by the National Bureau of Statistics [13].

In our model, each cycle corresponds to the 21-day treatment period as observed in the clinical trial, and the analysis is conducted over a 5-year timeframe. The primary outcome measures considered in our model include life year (LY), quality-adjusted life years (QALYs), and incremental cost-effectiveness ratios (ICERs). Consistent with the China Guidelines for Pharmacoeconomic Evaluations (2020) [14], we applied a discount rate of 5% and a willingness-to-pay (WTP) threshold 3 times the China’s GDP per capita in 2022, equivalent to US $38223.34/QALY) [15]. Additionally, we employed a half-cycle correlation for cost and survival estimates.

### Costs and utility input

In this study, we focused solely on direct medical expenses, including drug costs, follow-up tests, terminal care costs (TCC), and the management of serious adverse effects (SAEs). The costs of pembrolizumab, capecitabine, gemcitabine, and docetaxel in China were obtained from the Shandong drug centralized procurement platforms. The actual charging standards of local medical institutions were used for follow-up, while other cost data were derived from relevant literatures. All costs were presented in United States dollars and based on the 2022 exchange rate of 6.73 RMB/USD.

The analysis encompassed the costs associated with anemia and neutrocytopenia, specifically focusing on grades 3/4 AEs for which patients in the KEYNOTE-122 showed notably distinct probabilities. To streamline our model, we made the simplifying assumption that these adverse events were independent and maintained constant probabilities over the span of 5 years. Discontinuations resulting from SAEs were not taken into consideration. In our base case analysis, we assumed that all grade 3 and above AEs occurred within the initial 4 weeks of treatment initiation. The cost associated with AEs was determined by multiplying the estimated incidence rate of each event by its corresponding unit treatment cost. Additionally, we assumed that all patients underwent regular laboratory testing, encompassing a complete blood count (CBC), serum biochemical index (SBI), and imaging examination, including computed tomography (CT), nuclear magnetic resonance (NMR), and B-scan ultrasonography. The cost of TCC was factored into the final state. Utilities for patients with NPC were derived from a study conducted by Jiaqi Han [16], where the base values for PFS and PD were established as 0.76 and 0.35, respectively. A succinct summary of all pertinent information can be found in Table 1.

**Table 1:** Model parameters: baseline values, ranges, and distributions for sensitivity analysis

Parameter	Expected Value	Range	Distribution	Source
**Drug costs ($)**				
Pembrolizumab/cycle	5327.90	4262.32-5327.90	gamma	local charge
Capecitabine/cycle	36.39	29.11–36.39	gamma	local charge
Gemcitabine/cycle	44.16	35.33–44.16	gamma	local charge
Docetaxel/cycle	31.22	24.98–31.22	gamma	local charge
**AEs costs ($)**				
Anemia	6562.68	5250.14-7875.22	gamma	[16]
Neutrocytopenia	475.32	380.26-570.38	gamma	[16]
**Follow up monitoring cost ($)**				
Imaging/Surveillance	207.25	165.80-248.7	gamma	local charge
Laboratory test	11.89	9.51–14.27	gamma	local charge
Terminal care cost	1460.30	109.23-1825.38	gamma	[16]
**Utility**				
PFS	0.76	0.61–0.91	beta	[16]
PD	0.35	0.28–0.42	beta	[16]
**Probabilities, %**				
**Pembrolizumab**				
Anemia	0.90		beta	[9]
Neutrocytopenia	0.00		beta	[9]
**Bicalutamide**				
Anemia	10.70		beta	[9]
Neutrocytopenia	27.70		beta	[9]
Discount (%)	5.00	0 ~ 8	beta	[17]

AE, Adverse event; PFS, progression-free survival; PD, progressed disease

### Sensitivity analysis

We employed TreeAge Pro 2022 software to conduct sensitivity analyses, thereby validating our model. This rigorous approach enabled us to assess the robustness of model when confronted with variations within a reasonable range. To comprehensively evaluate the impact of parameter fluctuations on the ICER, we employed both one-way analysis and probabilistic sensitivity analysis (PSA). In the one-way sensitivity analysis, we meticulously examined the influence of individual parameters on the ICER by systematically varying them within the lower and upper limits derived from the 95% confidence intervals (95% CIs) or introducing a ± 20% alteration from the base case value [18]. In the PSA, we performed 1000 Monte Carlo simulations based on the presumed statistical distribution of parameters. Specifically, we employed gamma distributions for cost data, whereas beta distributions were adeptly applied for utility and incidence data. A comprehensive overview of the ranges and distributions of the parameters employed in the sensitivity analysis is meticulously summarized in Table 1. Drawing upon the data accumulated from the 1000 iterations, we derived a cost-effectiveness acceptability curve to effectively gauge the likelihood of pembrolizumab being deemed cost-effective across various levels of WTP levels for health gains (QALYs).

### Scenario analysis

To ensure the robustness of our model’s conclusions, we performed a range of scenario analyses. Firstly, as part of efforts to improve affordability and accessibility of anti-cancer medications, a philanthropic drug donation program for pembrolizumab has been established. This initiative allows patients who self-fund two courses of pembrolizumab treatment to receive an additional two courses of donated medication. Furthermore, patients who continue to self-fund two subsequent treatment courses are eligible to receive pembrolizumab as a gift until they either reach the PD status or complete 35 treatment cycles. We consider the participation of NPC in our study within this drug donation policy, and the cost of the treatment plan is then calculated based on the actual payments made by the patients after factoring in the drug donation. Secondly, we explored the potential impact of relevant drug policies in China on the costs of pembrolizumab. Pembrolizumab was approved relatively late in China and is currently not covered by medical insurance. However, in accordance with China’s relevant policies, the drug has the potential to participate in the national drug negotiation process. Taking into account the average price decrease of over 60% observed for anti-cancer drugs in the past three years, we propose simulating a scenario where the drug price is reduced by 60% as our second scenario. Finally, we investigated the effect of discounts (3% or 8%) on pharmacoeconomic results, as outlined in the China Guidelines for Pharmacoeconomic Evaluations (2020). All data analyses were conducted using TreeAge Pro 2022.

## Results

### Base-case analysis

The 21-day therapy costs were as follows: pembrolizumab ($5327.90), capecitabine ($36.39), gemcitabine ($44.16), and docetaxel ($31.22) (Table 1 2). We adopted ICER to access the cost-effectiveness of two treatment strategies. Compared with chemotherapy, pembrolizumab provided an additional 0.09 QALYs (approximately 1.08 quality-adjusted life-month) and 0.03 LYs (approximately 0.36 life-month), at an incremental cost of $36976.41. Our study revealed that pembrolizumab had an ICER (LY gained) of $1232547.00 and an ICER (QALY gained) of $422535.53 when compared with chemotherapy(Table 2). These figures surpassed the WTP threshold, indicating that pembrolizumab was a less cost-effective therapy in our study.

**Table 2:** Base results of pembrolizumab versus chemotherapy

Strategy	Chemotherapy	Pembrolizumab
Cost	$2734.55	$39710.96
Overall LYs	3.93	3.96
QALYs	2.73	2.82
Incr Cost		$36976.41
Incr Eff (LYs)		0.03
Incr Eff (QALYs)		0.09
ICER(USD per additional LY gained)		$1232547.00
ICER(USD per additional QALY gained)		$422535.53

LYs, life years; QALYs, quality-adjusted life years; Incr, incremental ratio; Eff, effectiveness; ICER, incremental cost-effectiveness ratio

### Sensitivity analysis

**Fig. 2:**
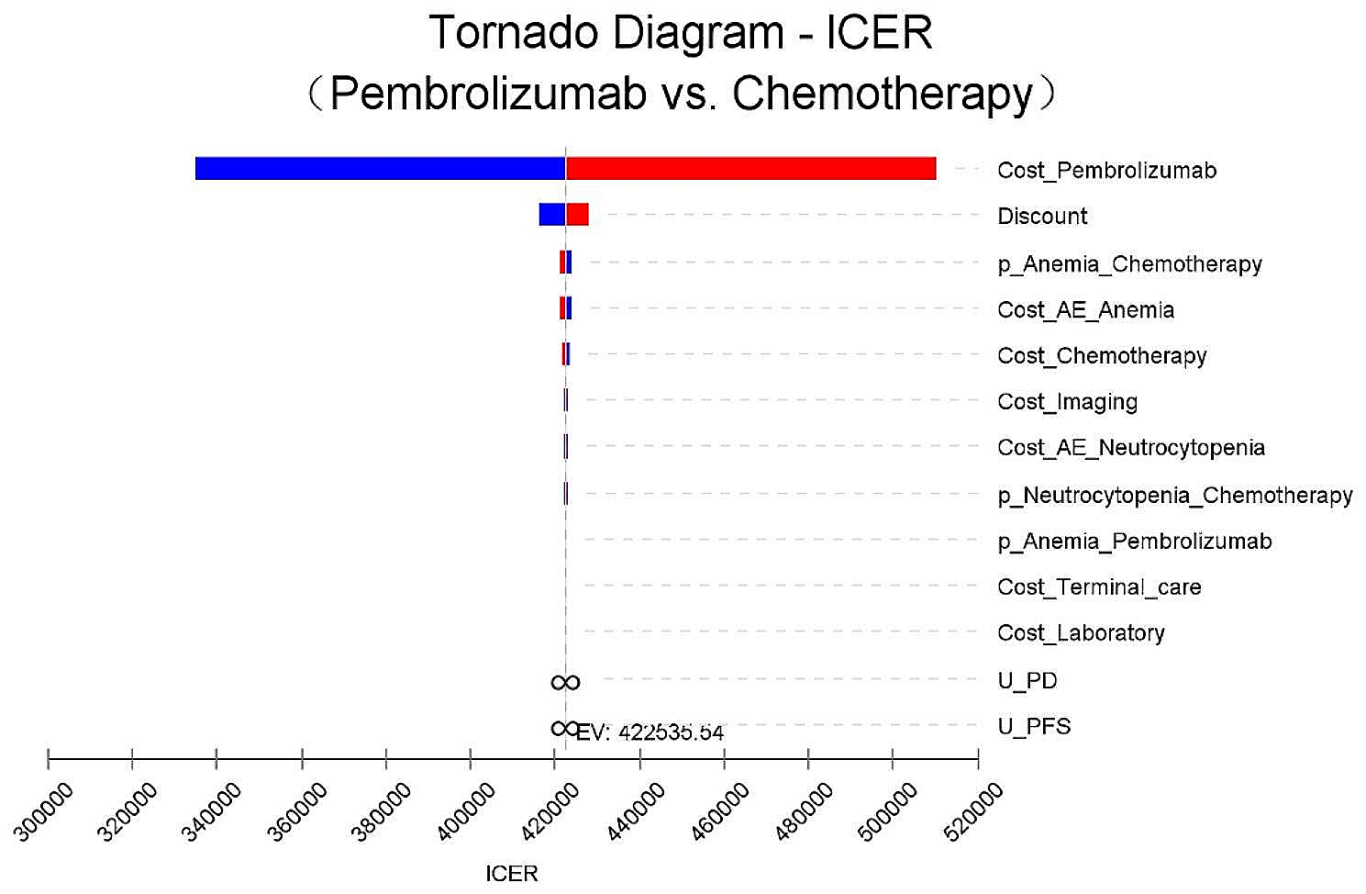
Tornado diagram of pembrolizumab versus chemotherapy in the one-way deterministic sensitivity analysis. P, probability; PD, progressed disease; PFS, progression-free survival; U, utility

The tornado diagram as shown in Fig. 2 illustrates an insightful sensitivity analysis of the ICERs comparing pembrolizumab with chemotherapy. Among the factors considered, the cost of pembrolizumab emerged as the most influential, closely followed by discounts. Notably, when the cost of pembrolizumab escalated to the higher threshold range ($4262.32–$6393.48), the ICER far exceeded the acceptable WTP threshold of $38223.34 per QALY. These findings underscore the robustness of results, as even the pivotal variable of pembrolizumab price failed to overturn the outcomes. On the other hand, other variables, including the probability of AEs, costs associated with adverse reaction treatment, chemotherapy, and imaging, as well as the utility of PFS and PD, exerted only mild or moderate effects on the ICER.

**Fig. 3:**
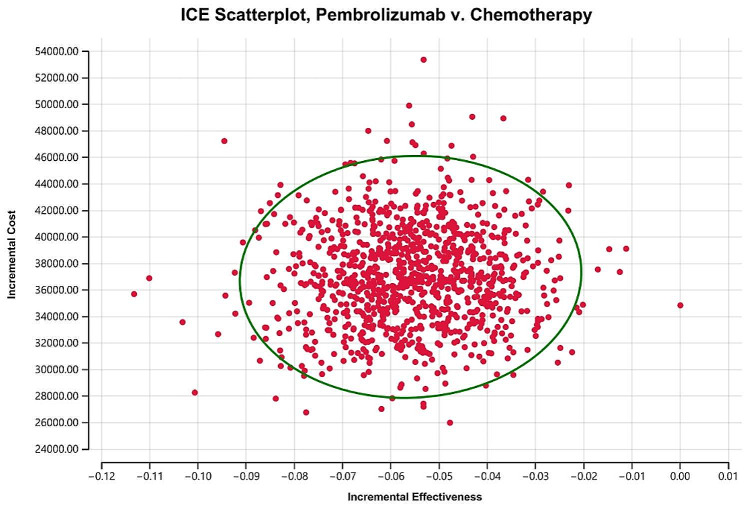
1000 Monte Carlo simulation diagram of pembrolizumab versus chemotherapy in the probabilistic sensitivity analysis. ICE, incremental cost-effectiveness

The findings from our Monte Carlo simulation have unveiled noteworthy insights regarding the cost-effectiveness of chemotherapy and pembrolizumab. The average cost and efficacy of chemotherapy were determined to be $2729.09 ± $144.66 and 1.64 ± 0.12 QALY, respectively. In stark contrast, pembrolizumab exhibited substantially higher average costs and lower efficacy, standing at $39738.34 ± $3729.48 and 1.58 ± 0.13 QALY, respectively. Further analysis of these results using a probabilistic sensitivity analysis (PSA) suggested that pembrolizumab was unlikely to be considered a cost-effective option when compared to chemotherapy. At a WTP threshold of $38223.34 per QALY, pembrolizumab exhibited a 0% probability of being deemed cost-effective (as depicted in Fig. 3). Given these compelling findings, it becomes evident that pembrolizumab may not be the most efficient or cost-effective treatment alternative for patients grappling with platinum-pretreated, recurrent or metastatic NPC.

### Scenario analysis

The scenario analysis outcomes are comprehensively presented in Table 3. In the philanthropic drug donation program scenario, it was observed that the ICER witnessed a substantial decrease of $34,754.72, falling well below the WTP threshold. However, when considering a 60% reduction in the price of pembrolizumab, the resultant ICER ($159,516.60) in comparison to chemotherapy surpassed the WTP threshold of three times the GDP per capita. Furthermore, when adjusting the discount rates to 3% and 8% respectively, the ICER for pembrolizumab versus chemotherapy amounted to $410,285.33 and $439,752.11 correspondingly. These findings unequivocally indicate that pembrolizumab emerges as the less cost-effective treatment option at both discount rates. It is notable that discounts have a modest impact on the results, and only significant reductions in drug prices render pembrolizumab economically viable.

**Table 3:** Scenario analysis results

Scenario	Strategy	Cost	Incr Cost	Eff	Incr Eff	ICER	Remarks
1	Chemotherapy	2734.55		2.73			philanthropic drug donation program
	Pembrolizumab	5775.96	3041.41	2.82	0.09	34754.72	
2	Chemotherapy	2734.55		2.73			price dropped by 60%
	Pembrolizumab	16693.97	13959.43	2.82	0.09	159516.66	
3	Chemotherapy	2802.98		2.88			3% discount
	Pembrolizumab	40167.86	37364.88	2.97	0.09	410285.33	
	Chemotherapy	2646.93		2.55			8% discount
	Pembrolizumab	39086.87	36439.94	2.63	0.08	439752.11	

Incr, incremental ratio; Eff, effectiveness; ICER, incremental cost-effectiveness ratio

## Discussion

The predominant approach for managing recurrent and metastatic NPC currently revolves around palliative systemic chemotherapy. However, the field of systemic treatment for recurrent or metastatic NPC lacks comprehensive clinical research, resulting in chemotherapy regimens primarily consisting of platinum-containing dual or triple drug regimens for non-nasopharyngeal squamous cell carcinoma of the head and neck [19, 20]. Notably, ICIs have gained considerable attention owing to their remarkable therapeutic efficacy in various solid tumors, revolutionizing specific domains of oncology over the past decade. Patients can now receive single-agent therapy with anti-CTLA-4 (tremelimumab or ipilimumab), anti PD-1 (nivolumab or pembrolizumab), or anti PD-L1 (avelumab, atezolizumab or durvalumab), or a combination of anti CTLA-4 and anti PD-1 [21]. Several clinical studies have established the effectiveness and safety of pembrolizumab in treating relapsed or refractory NPC. Findings from the KEYNOTE-028 study suggest that pembrolizumab monotherapy may be effective for advanced NPC, while maintaining manageable safety profiles [10]. In the CAPTAIN-1ST study and JUPITER-02 study, a standard chemotherapy regimen was combined with immunotherapy for advanced first-line NPC [22, 23]. The camrelizumab and teriprizumab groups exhibited significantly prolonged PFS, surpassing the placebo group in terms of efficacy. Extensive research results indicate that the combination of ICIs with chemotherapy or targeted therapy can yield synergistic effects, potentially influenced by chemotherapy and targeted therapy on tumor neoantigen exposure and the tumor immune microenvironment [24].

Carcinoma poses a substantial health burden in China, prompting a comprehensive cost-benefit analysis of NPC treatment in the country. Multiple studies have scrutinized the expenses associated with managing recurrent or metastatic NPC. Regarding the cost-benefit analysis of locally advanced NPC treatment, Fei et al. discovered that incorporating nimotuzumab into the standard treatment regimen yields significant survival benefits, albeit at a less cost-effective outcome [25]. Conversely, She et al.’s investigation revealed that metronomic capecitabine, employed as adjuvant chemotherapy, represents a cost-effective strategy [26]. In terms of cost-effectiveness, metronomic capecitabine outperforms nituzumab. In the realm of induction chemotherapy, two cost-benefit analyses juxtaposed gemcitabine and cisplatin (GP) against docetaxel and cisplatin plus fluorouracil (TPF). Wu’s research indicates that GP, compared to TPF, exhibits an ICER of $2804.44 per QALYs [27]. Similarly, Yang’s study demonstrated that the GP regimen yielded an additional 0.42 QALY with an incremental cost of $3821.99, resulting in an ICER of $9099.98 per QALY [28]. Despite the higher overall cost of the GP scheme in both literatures, it shows superior survival benefits. Chen et al.’s article highlights the cost-effectiveness of gemcitabine plus cisplatin compared to fluorouracil plus cisplatin in treating recurrent or metastatic NPC, with the total cost of GP amounting to $17,920 [29]. Drawing upon the analysis of two phase III clinical trials, the combined cost of tereprimab and GP reaches $48,525, demonstrating greater cost-effectiveness than carelizumab plus GP chemotherapy [30, 31]. However, there is a lack of research evaluating the cost-effectiveness of pembrolizumab compared to chemotherapy for platinum-pretreated, recurrent or metastatic NPC patients in China.

Our analysis indicates that pembrolizumab is less cost-effective than chemotherapy for platinum-pretreated, recurrent or metastatic NPC patients, with ICER of $1232547.00 per additional LY and ICER of $422535.53 per additional QALY. These figures far exceed the WTP threshold in China. Sensitivity analyses confirmed the robustness of our findings, as no changes affected the overall outcome. Among the parameters examined, the price of pembrolizumab emerged as the most sensitive factor compared to chemotherapy. Discount rates also played a significant role in determining cost outcomes. Even after adjusting the discount rate to 3% or 8%, the ICER for pembrolizumab remained above the WTP threshold. PSA results showed a low probability (well below 0%) of pembrolizumab being cost-effective compared to chemotherapy.

To improve accessibility to medications, the Chinese government has implemented the National Reimbursement Drug List Negotiation (NRDLN) since 2016 [32]. Recent drug negotiation policies in China have led to significant price reduction of up to 60% for biological agents. The national centralized procurement policy has also resulted in a substantial price reductions of chemotherapy drugs, up to 80% and 90%. In this study, we utilized the centralized procurement price of chemotherapy drugs in Shandong Province, which contributes to pembrolizumab’s lack of cost-effectiveness. Pembrolizumab has not been included in the medical insurance and drug negotiations, leaving ample room for price reduction. Although pembrolizumab does not offer a high QLAY value, it effectively reduces adverse reactions and improves patients’ quality of life, providing advantages for its use in NPC treatment. Pharmacoeconomic evaluation can also serve as a reference for its clinical application and inclusion in medical institution directories.

There are certain limitations to be considered in this study. Firstly, the analyses were primarily based on data from the KEYNOTE-122 trial, which is currently the only randomized phase III trial comparing pembrolizumab to chemotherapy in NPC patients. Therefore, any biases inherent in that trial may have influenced the results of this study. Secondly, although the KEYNOTE-122 trial reported no significant improvement in OS, it did demonstrate a greatly improvement in safety. But this study did not consider the negative effects caused by adverse reactions. Thirdly, the actual costs of drugs and examination fees used in this study were based on local charges, and there may be variations in fees charged by medical institutions in various provinces in China. Therefore, the finding may not present the national average situation and can only reflect the specific circumstances of certain departments and regions. Moreover, a detailed hierarchical modeling discussion on gene deletion in the population and subgroup analyses were not conducted, making it challenging to simulate changes in the disease and hierarchical treatment. Nonetheless, despite these limitations, this research holds great significance within China’s healthcare system and clinical practice.

## Conclusion

Our results indicate that pembrolizumab is less cost-effective compared to chemotherapy, with an estimated cost-effectiveness profile falling within a range of $12,741.11297 to $38,223.34 per QALY at the WTP threshold. Although pembrolizumab may not meet the standard cost-effectiveness criteria, these findings can still serve as valuable evidence to guide discussions on drug pricing and inform informed decision-making when selecting appropriate treatment options.

The funder had no role in the design and conduct of the study; collection, management, analysis, and interpretation of the data; preparation, review, or approval of the manuscript; and decision to submit the manuscript for publication.

### Electronic supplementary material

Below is the link to the electronic supplementary material.

Supplementary Material 1

Supplementary Material 2

## Data Availability

Data will be made available.
